# A novel multimodal needs assessment to inform the longitudinal education program for an international interprofessional critical care team

**DOI:** 10.1186/s12909-022-03605-2

**Published:** 2022-07-13

**Authors:** Heyi Li, Yuqiang Sun, Amelia Barwise, Wenjuan Cui, Yue Dong, Aysun Tekin, Qingzhong Yuan, Lujun Qiao, Ognjen Gajic, Alexander Niven

**Affiliations:** 1 Department of Medicine, Division of Pulmonary and Clinical Care Medicine, Mayo Clinic, Rochester, MN USA; 2 Department of Emergency Medicine, China Medical University, Shenyang, China; 3 Department of Anesthesiology and Perioperative Medicine, Mayo Clinic, Rochester, MN USA; 4 Department of Intensive Care Medicine, Shengli Oilfield Central Hospital, Dongying, China

**Keywords:** Medical continuing education, Medical training, Learning needs assessment, Critical care, Intensive care, Entrustable professional activity, Curricular milestones, Delphi, Q method

## Abstract

**Background:**

The current global pandemic has caused unprecedented strain on critical care resources, creating an urgency for global critical care education programs. Learning needs assessment is a core element of designing effective, targeted educational interventions. In theory, multimodal methods are preferred to assess both perceived and unperceived learning needs in diverse, interprofessional groups, but a robust design has rarely been reported. Little is known about the best approach to determine the learning needs of international critical care professionals.

**Method:**

We conducted multimodal learning needs assessment in a pilot group of critical care professionals in China using combined quantitative and qualitative methods. The assessments consisted of three phases: 1) Twenty statements describing essential entrustable professional activities (EPAs) were generated by a panel of critical care education experts using a Delphi method. 2) Eleven Chinese critical care professionals participating in a planned education program were asked to rank-order the statements according to their perceived learning priority using Q methodology. By-person factor analysis was used to study the typology of the opinions, and post-ranking focus group interviews were employed to qualitatively explore participants’ reasoning of their rankings. 3) To identify additional unperceived learning needs, daily practice habits were audited using information from medical and nursing records for 3 months.

**Results:**

Factor analysis of the rank-ordered statements revealed three learning need patterns with consensual and divergent opinions. All participants expressed significant interest in further education on organ support and disease management, moderate interest in quality improvement topics, and relatively low interest in communication skills. Interest in learning procedure/resuscitation skills varied. The chart audit revealed suboptimal adherence to several evidence-based practices and under-perceived practice gaps in patient-centered communication, daily assessment of antimicrobial therapy discontinuation, spontaneous breathing trial, and device discontinuation.

**Conclusions:**

We described an effective mixed-methods assessment to determine the learning needs of an international, interprofessional critical care team. The Q survey and focus group interviews prioritized and categorized perceived learning needs. The chart audit identified additional practice gaps that were not identified by the learners. Multimodal methods can be employed in cross-cultural scenarios to customize and better target medical education curricula.

**Supplementary Information:**

The online version contains supplementary material available at 10.1186/s12909-022-03605-2.

## Introduction

Critical care professionals need continuing education to sustain their competence in a broad range of knowledge, skills, and attitudes demanded by subspecialty practice. They face challenges incorporating new evidence-based practices that continue to emerge at a rapid pace. Their education needs are particularly urgent in emerging intensive care settings in economically developing countries [1], prompting the World Health Organization (WHO) and international subspecialty societies to advocate for increased education programs in these areas [2, 3].

To facilitate timely and accurate delivery of best practice delivery in critically ill patients, a group of international critical care physicians and researchers developed the Checklist for Early Recognition and Treatment of Acute Illness and Injury (CERTAIN) program [4, 5], a structured approach to critically ill patients. The CERTAIN study group has provided interprofessional, competency-based training for more than a thousand intensive care physicians and nurses in more than 50 countries, and demonstrated improved adoption of evidence-based best practices in 36 intensive care units in 15 different countries [6, 7]. Based on a longitudinal pilot intervention that demonstrated successful integration of CERTAIN practices in an intensive care unit (ICU) in Bosnia and Herzegovina, the investigators designed a longitudinal education and quality improvement program (Knowledge Translation into Practice, KTIP) targeting an international audience. Our goal is to maximize the impact of this intervention by customizing the curriculum to individual learning needs [8].

There have been considerable efforts over the past decade to strengthen the instructional design, delivery, and outcomes measurement of continuing medical education programs. A well-conducted needs assessment is considered a core contributor to the success of the educational program [9]. Systematic reviews have shown that programs predicated on a well-designed needs assessment are more effective in changing physician behaviors [10, 11]. However, robust learning needs assessment models are rarely reported in the literature, and there is little agreement on how to measure learning needs among international healthcare professionals [12]. This is in part due to the various possible states of self-knowledge commonly described using the Johari window (Supplemental Table 1) [13]. Learning needs within the Johari window framework can be classified as perceived or unperceived. Qualitative methods, such as informal discussions, questionnaires, or structured interviews are often used to invite the learners to express their perceived learning needs. However, learners may remain ‘blind’ to their unperceived learning needs despite these activities. As Sibley et al. observed, medical practitioners tend to pursue education around topics in which they excel, while avoiding areas in which they are deficient [14]. Quantitative methods, such as chart audits, tests, or direct observation of practice habits, are required to reveal unperceived learning needs (Supplemental Table 2) [15–17]. For learning needs assessment to be robust, it should use mixed techniques combining qualitative and quantitative data from a probabilistic sample that includes employees with diverse roles and different skill and experience levels [18]. Unlike previous studies that were predominantly survey-based [12], this study proposed a novel learning needs assessment process using mixed methods and implemented it in a pilot group of international critical care professionals.

First described in 1953 by psychologist and physicist William Stephenson, Q method is a systematic, semi-quantitative study of subjectivity [19]. Different from traditional surveys that provide a summary of opinions, Q method categorizes participants and identifies consensual and divergent opinions within a study population using by-person factor analysis [20, 21]. It has been used frequently in medical settings to identify physicians’ and nurses’ learning preferences [22–24]. In the field of critical care, the learners are often a heterogeneous group of physicians, nurses, and other medical professionals. Thus, Q method was ideal to identify the typology of learners’ needs. Using the Q method to divide learners into subgroups, we subsequently conducted focus group interviews with each subgroup to investigate the rationale behind their identified learning priorities, as well as structured chart audits to identify unperceived learning needs. The three combined stages add up to a novel learning needs assessment model that is different from the previously reported single-method models.

## Methods

### Study subjects

In 2020, the CERTAIN investigators agreed to provide remote, structured longitudinal training using the KTIP program at Shengli (Victory) Oilfield Central Hospital in Dongying, Shandong, China. Dongying is a coastal city with a population of nearly two million. The large public community hospital has 1891 beds, including a mixed 27 bed ICU. Twenty-four critical care professionals participated in the training programs, including 20 physicians and 4 nurses. There were 8 female and 16 male participants. Their demographic features are listed in Table 1. All participants have bachelor’s or master’s degrees.

**Table 1:** Participants of the Q survey and their correlation with the factors (subgroups)

Profession	Participant number	Teaching responsibilities	Subgroup (Factor analysis)
**Physician**	I	Yes	1*
	II	No	1*
	III	Yes	2*
	IV	No	2*
	V	No	2*
	VI	Yes	2
	VII	No	3
**Nurse**	VIII	Yes	1*
	IX	No	2
	X	No	3
	XI	No	3*

*p < 0.05. *P* value was generated by factor analysis

Prior to the start of training, a convenience sample of seven physicians and four nurses was selected to participate in a learning needs assessment. The team’s daily medical and nursing records were also audited for 3 months to identify unperceived learning needs. Oral consent and written agreement for training activities was obtained from all participants. The study was approved by the Mayo Clinic Institutional Review Board (20–007896) and the Ethics Committee at Shengli Oilfield Central Hospital (Q/ZXYY-ZY-YWB-LL202039).

### Multimodal learning needs assessments

**Fig. 1:**
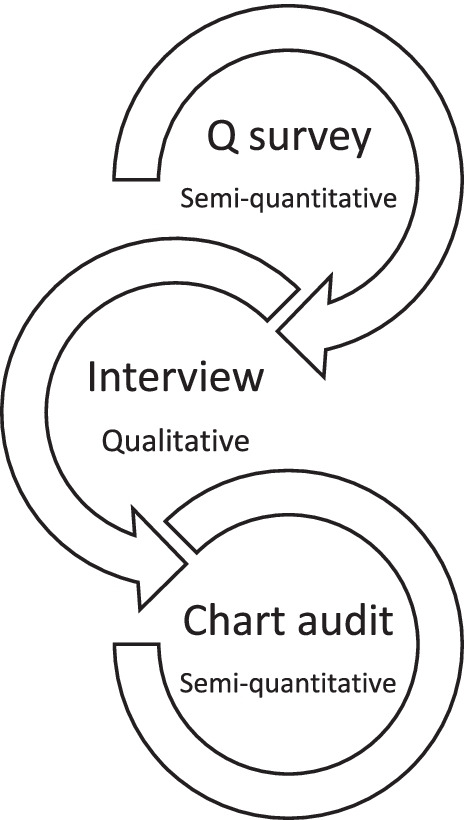
Structured design of leaning needs assessments

To design a multimodal learning needs assessment process that can be used for diverse international critical care groups, we chose a combination of the Q method survey, focus group interviews, and chart audits as the basis of our learning needs assessment model (Fig. 1).

### Phase 1. Preparation of Q set

The first step in the Q method was to generate a set of statements (Q set) describing essential critical care performance elements that would be reviewed and ranked by the learners later. The investigators chose to describe these core performance elements in the form of entrustable professional activities (EPAs). EPA is a common conceptual tool in competency-based graduate medical education. Each EPA is an independently executable, observable, and measurable task or responsibility to be entrusted to the unsupervised execution by a trainee once he or she has attained sufficient specific competence [25, 26].

The investigators reviewed and compared published literature on critical care educational objectives and existing critical care curricula, such as American board certification blueprints [27, 28], American critical care training programs(i.e. Fundamentals of Critical Care Support Course, offered by the Society of Critical Care Medicine) [29], Accreditation Council for Graduate Medical Education (ACGME) Reporting Milestones [30], TeamSTEPPS® curriculum [31], and the ACGME Clinical Learning Environment Review Pathways to Excellence [32]. Then the investigators drafted 40 candidate statements using the ACGME Reporting Milestones as a guide to content selection and compared them to published Chinese critical care competency standards [33] to ensure all relevant content domains were considered. Each statement provided the description of a critical care EPA.

The list of EPA statements was next refined and narrowed by a second group of critical care educational experts (*n* = 5) using Delphi method. These experts were all program directors of critical care fellowship programs. They worked independently from the authors of this study. During the first Delphi round, participants were asked to independently rate each of the 40 EPAs using the question “I would like to include this EPA in a list describing the critical care activities demonstrated at the end of this training program” on a 5-point Likert scale. During the second Delphi round, the same group of participants received the same rating sheet with their individual round one rating, and the distribution of other group members’ ratings with calculated mean and median. From there, participants were asked to independently re-rate each EPA for the same question as round one, answering yes/no for each individual EPA to select a total of 20 EPAs to include in the final list. The second round was repeated until a consensus was achieved on a final list of 20 EPAs.

### Phase 2.1 Q method survey

The Q method survey was distributed to Chinese participants via an online webpage using HTMLQ. The participants were asked to rank a set of digital cards, each with a single EPA, into ‘more important’, ‘less important’, and ‘neutral’ docks. Then they were asked to place the cards onto a pre-defined grid associated with an anchored scale based on their perceived learning priorities (Supplement Fig. 1). Each participant’s ranking pattern was transformed into an array of numerical data according to the grid in which each statement was placed. The statement that was placed at the ‘most important’ end of the distribution received a score of + 4, the next two statements received + 3, the next two statements received + 2, and so forth, all the way down to the statement that was considered ‘least important’, which received a score of − 4. Statements placed in the middle of the grid were assigned scores of 0. All participants’ arrays of numerical data formed a correlation matrix, from which a set of ‘factors’ were extracted. Each factor represented a cluster of similar ranking patterns. The factor analysis also identified key consensual or divergent opinions that shaped the patterns of opinions. The data analysis was performed on Ken-Q analysis, a web application of Q methodology (Shawn Banasick, 2019, Version 1.0.6) [34].

### Phase 2.2 post survey interview

The factor analysis identified learners who had similar ranking patterns and thus categorized them into subgroups. Focus group interviews with the participants were conducted in their native language, Mandarin Chinese. Participants were invited to review their individual ranking and the common ranking pattern shared by the subgroup. Then the investigator asked them to describe the reasoning behind their ranking choices. The interviews were recorded and transcribed into English for further qualitative review. The transcription was reviewed by two investigators to identify keywords and concepts using a thematic analysis approach.

### Phase 3 chart audit

Medical and nursing records were reviewed for learning needs assessment over a three-month consecutive period prior to the start of the KTIP program simultaneously as the participants go through the surveys described in phase 2. All adult patients who were admitted to the ICU for critical illness were included. The charts of the sampled patients were audited at the time of admission, then on day 0, day 1, day 2, day 3, day 7, day 14, day 21, and day 28, if documentation was available on that date. Data were de-identified and documented in a series of care process documentation sheets based on the framework developed by the United States Institute of Health Care Improvement [35].

The investigators focused on metrics that reflected adherence to commonly accepted best critical care and patient-centered care practices. For recommended daily best practices, incidence rates of non-adherence were calculated using the number of observed non-adherence events divided by the total observation days.

## Results

### Leaning need assessment participant baseline characteristics

Eleven critical care professionals from a mixed medical/surgical/cardiac ICU formed the convenience sample group for the perceived learning need assessment, including 4 nurses and 7 physicians (Table 1). Three participants were male. Their mean age was 38.5 years (standard deviation: 4.9 years). They had 10.9 years (standard deviation: 4.0 years) of clinical critical care experience.

### Q method survey

A Q set of 20 EPA statements was generated by critical care education experts after a two-round, one-cycle Delphi process (Table 2). The statements covered five essential domains of critical care practice: organ support and disease management (13 statements), practical skills (2 statements), quality improvement (1 statement), patient-centered care and communication (1 statement), and interprofessional skills (3 statements).

**Table 2:** Q set: 20 EPA statements generated by critical care education experts

Domains of critical care practice		Statements^a^
Organ support and disease management	1	Evaluate and manage patients presenting with acute respiratory failure, including early recognition, diagnostic evaluation, and treatment of most likely causes including pneumonia, obstructive lung disease exacerbation, congestive heart failure, pulmonary embolism and tension pneumothorax.
	2	Evaluate and manage patients with sepsis and septic shock, including early recognition, resuscitation, appropriate antibiotics, and systematic evaluation for source control.
	3	Evaluate and manage common nephrology conditions in the ICU, including acute kidney injury, renal replacement therapy, and acid base and electrolyte disorders.
Patient-centered care and communication	4	Provide compassionate, patient-centered care, engaging with patients and family members in shared decision making using collaborative communication skills, empathy, and respect.
Organ support and disease management	5	Evaluate and manage patients presenting with acute common cardiovascular conditions, including arrhythmias, acute coronary syndromes, valvular heart disease, congestive heart failure, and vascular emergencies.
	6	Evaluate and manage patients with shock, including early recognition, rapid diagnostic evaluation, and targeted treatment of cardiogenic, hypovolemic/hemorrhagic, distributive and obstructive shock, including targeted vasopressor management.
	7	Evaluate and manage patients presenting with poisoning or overdose, including complications of alcohol, drug intoxication and withdrawal.
	8	Evaluate and manage common gastroenterology conditions in the ICU, including acute gastrointestinal hemorrhage, difficile colitis, bowel obstruction and perforation, complications of hepatobiliary disease, and pancreatitis.
	9	Evaluate and manage common hematology and oncology conditions in the ICU, including coagulopathy, acute / massive hemorrhage, common malignancies and their associated complications.
	10	Evaluate and manage common neurologic conditions in the ICU, including encephalopathy, seizure, stroke, and intracranial hemorrhage.
	11	Identify, evaluate and manage patients with ARDS^b^, collaborating with Respiratory Therapy and utilizing institutional protocols to deliver safe and effective lung protective ventilation, rapidly identify patients with refractory hypoxemia, and appropriately employ early liberation strategies.
Procedure/resuscitation skills	12	Resuscitate and stabilize critically ill patients, performing necessary diagnostic and therapeutic interventions in a timely manner and effectively coordinating care with the interprofessional critical care team and appropriate consultants.
	13	Safely and efficiently perform procedures common to the practice of critical care medicine, and demonstrate understanding of indications, contraindications, limitations, and complications of these interventions.
Organ support and disease management	14	Diagnose and manage acute pain in critical illness and the perioperative setting, including appropriate use of opioids, non-opioid analgesics, and assessment scales.
	15	Evaluate and manage common critical care infections, including meningitis/encephalitis, pneumonia, catheter related bloodstream infections, simple and complicated biliary, urinary tract, skin and soft tissue infections, and opportunistic pathogens commonly seen in immune compromised hosts.
Interprofessional skills	16	Professional, respectful and timely in the execution of all clinical activities, with appropriate communication and collaboration within interprofessional team.
	17	Ensures effective transitions of care through consistent, concise communication of patient care plans and recommendations.
	18	Leads efficient and effective ICU rounds by soliciting and incorporating collaborative input from the interprofessional team, appropriate consulting services, patients and families to develop a well-organized, appropriate plan of care.
Quality improvement	19	Efficiently employ critical care protocols and checklists to prevent common critical care complications, and effectively diagnose and manage delirium, venous thromboembolism, nosocomial infections, malnutrition, hyperglycemia, decubitus ulcers, and musculoskeletal complications.
Organ support and disease management	20	Evaluate and manage perioperative patients and common post-surgical complications.

^a^ The statements are ordered from highest to lowest priority based on modified Delphi rankings. The statements covered five essential domains of critical care practice: organ support and disease management (statement 1,2,3,5,6,7,8,9,10,11,14,15,20), practical skills [12, 13], quality improvement [19], patient-centered care and communication [4], and interprofessional skills [16–18]

^b^ *ARDS* Acute respiratory distress syndrome

Three subgroups were identified using the Q survey and factor analysis. Each subgroup was represented by a ‘factor’, a ranking list of the 20 EPAs that reflected the learning priorities expressed by the subgroup. Three participants demonstrated correlation with factor 1 (subgroup 1), five participants correlated with factor 2 (subgroup 2), and three participants (VII, X, XI) correlated with factor 3 (subgroup 3). Seven out of eleven participants’ correlation achieved statistical significance (*p* < 0.05) (Table 1).

All subgroups perceived high interest in several EPAs about organ support and disease management. For example, subgroups 1 and 2 ranked ‘Evaluate and manage patients with shock’ (statement 6) as the most important learning object, while subgroup 3 valued ‘Evaluate and manage perioperative patients’ (statement 20) most highly. All subgroups perceived moderate interest in quality improvement, low to moderate interest in interprofessional skills, and low interest in patient-centered communication. The three subgroups had different opinions on learning procedure/resuscitation skills. Subgroup 1 participants were not interested, however, subgroups 2 and 3 were moderately or highly interested (Table 3) in learning procedures or resuscitation skills.

**Table 3:** Interest in different categories of EPAs expressed by the participants

	Interest level (reflected by ranking of importance)		
EPA categories	Subgroup 1	Subgroup2	Subgroup3
Organ support and disease management	High	High	High
Quality improvement	Moderate	Moderate	Moderate
Interprofessional skills	Low to moderate	Low to moderate	Low to moderate
Patient-centered communication	Low	Low	Low
Procedure/resuscitation	Low to moderate	Moderate	Moderate to high

### Post survey interviews

The interviews were conducted in three subgroups. Thematic analysis identified a number of consensual or divergent concepts. The associated quotes are listed in Table 4.

1. Overall impression from the ranking activities: eager to learn

**Table 4:** Themes identified from the interviews and related quotes

**Eager to learn**	*“Everything attracted me. I would like to learn everything if time allows”. (II)* *“It was hard to pick from the cards. They were all important.” (III)*
**Organ support and disease management**	*“The most severe shock often necessitates more than two vasopressors. We are curious about the choice of medication, maximal dose, drug interaction, and how to simplify or de-escalate.” (VII)* “I hope to learn about advanced life-support techniques, like ECMO*.” (II) *“How do you assess one’s ability to cough? Many patients cannot manage their secretions after extubation, despite having rehabilitation.” (V)* *“For patients with multi-organ failure, we come across many nutritious problems like diarrhea, ileus, and poor absorption of enteral feeding. How do we assist the recovery of the digestive tract?” (VIII)* *ECMO: extracorporeal membrane oxygenation
**Quality improvement**	*“Our goal is to create standardized, protocolized workflow. We have access to the guidelines, but how to implement guidelines into daily practice? How to engage all colleagues to adhere to best practices instead of being guided by personal experience alone?” (VII)* *“The patients will benefit from standardized care.” (IV)* *“In many rural hospitals from where our patients are transferred, the choice and duration of antibiotics are not ideal. Drug-resistant bacteria are common.” (I)*
**Interprofessional skills**	*“Standardized presentations on rounds are not mandatory as we are not a teaching hospital. We do not conduct typical multidisciplinary rounds. I feel confused when the consult team’s opinions are different from ours.” (III)* *“While co-managing patients with operative teams, occasional disagreement on medical assessment, such as the necessity of an intervention, or the patient’s readiness for extubation, leaves us in a hard situation.” (I)*
**Patient-centered communication**	*“My least interested topic was communicating with family. The patient-doctor relationship may be different in the US. I guess the way they communicate may be quite different, too.” (VII)* *“I also ranked the patient communication as a less important one because considering the cultural difference, it must be hard to adopt directly what the American doctors do. I care relatively more about the diagnostic and therapeutics.” (I)* *“Our patient engagement is limited. Some patients have little educational background. We are often asked (by family) to hide the cancer diagnosis from the patient. There are many barriers.” (VI)*
Procedure/resuscitation **skills**	*“I do all procedures comfortably except tracheostomy of high complexity. (VI)* *“We’re starting a critical care residency. The younger doctors need more training. We would like to learn about educational methods and training standards for procedures.*” *(I)*

Many participants explained that they were interested in all EPAs, including the ones they ranked as less important.

2) Organ support and disease management: strong interest

The learners expressed strong interest in refining their organ support techniques, especially for their sickest patients. Their needs were deeply rooted in their daily practice and extended to nuances of therapeutics. Five participants (I, II, III, V, VI) expressed that they were eager to learn more about Positive End Expiratory Pressure (PEEP) titration for refractory respiratory failure. Advanced life support, liberation from prolonged mechanical ventilation, and nutrition support for the critically ill were mentioned frequently by learners.

3) Quality improvement: moderate interest

Several participants expressed interest in standardizing and protocolizing commonly performed practices. ‘Flowcharts’ and ‘algorithms’ were desired. Interest in antibiotic stewardship was expressed by participant I.

4) Interprofessional skills: low to moderate interest

Although structured rounds are conducted daily, the clinicians lacked a template for sharing information on rounds. Some participants shared the need for a communication tool that assists multidisciplinary collaboration.

5) Patient-centered communication: Low interest

The participants explained that patient-centered communication was indeed considered important, but not as important as disease management. Some participants shared that the survey activity reminded them about the importance of patient engagement. Their main concern about conducting international education on patient-centered communication is that it may be less feasible than other educational efforts due to cultural and language barriers and differing cultural norms.

6) Procedure/resuscitation skills: variable interest

The opinions in regard of procedure/resuscitation skills were variable. The experienced physicians expressed confidence in performing procedures. However, the physician in charge of the residency program (participant I) pointed out needs existed among the doctors in training.

### Chart audit

We reviewed 101 patient charts during the audit period. Eighty-five patients had baseline information on the day of admission that is summarized in Supplemental Table 3.

One hundred and one patients had care process data available over a total of 436 observed days. Data detailing non-adherence incidence rates are summarized in Table 5. Adherence to deep vein thrombosis (DVT) prophylaxis was high in all patients. We also observed high adherence to oral hygiene, head of bed elevation, peptic ulcer prevention, and sedation discontinuation assessment in mechanically ventilated patients. The documented assessment rates of central line removal, urinary catheter removal, spontaneous breathing trial, and antimicrobial therapy discontinuation were suboptimal. Family discussions were documented infrequently.

**Table 5:** Adherence to best practice observed in a 3-month audit period

Best-practice	Patients (N)	Observed days (N)	Best-practice omission events (N)	Best practice omission incidence rate (number of events per 1000 days)
**General practice**	**101**	**436**		
DVT^a^ prophylaxis	101	436	20	45.9
Family discussion documentation	101	436	332	761.5
**Mechanical ventilation**	**45**	**170**		
Oral hygiene	45	170	5	29.4
Elevation of head of bed	45	170	1	5.9
Peptic ulcer prevention	45	170	13	76.5
Spontaneous breathing trial assessment	45	170	64	376.5
**Antimicrobial therapy and sedation**				
Sedation discontinuation assessment	44	131	24	183.2
Antimicrobial therapy discontinuation assessment	72	281	96	341.6
**Devices**				
Central line removal assessment	50	194	82	422.7
Urinary catheter removal assessment	94	390	155	397.4

^a^*DVT* Deep vein thrombosis

## Discussion

Although a needs assessment is a well-accepted element of instructional design, the best approach to define the learning needs of an international audience to inform the longitudinal delivery of a virtual critical care curriculum has not been described. In this study, we described a multi-stage, mixed-method learning needs assessment model that is different from any previously reported assessment tool. This approach enabled us to better understand not only the practice but also the cultural context of our learner group. The pilot group expressed strong interest in education on organ support and disease management topics, and moderate interest in quality improvement. Interest in interprofessional communication and patient-centered communication was relatively low, and interest in learning procedure/resuscitation skills was mixed. While chart audit demonstrated a high level of routine completion of many elements of evidence-based daily care, it also identified opportunities for improvement in discontinuation of invasive devices, assessing for spontaneous breathing trial, and antimicrobial therapy discontinuation. Communication with family was another potential underperceived learning need. This learning needs assessment model will enable us to develop a meaningful collaboration more quickly and deliver our virtual international education program more effectively.

Post-graduate critical care education in China is transitioning from a traditional to a competency-based model [33]. The first nationwide agreement about evaluation and accreditation of Chinese critical care trainees was published in 2016, to describe the minimum required competencies for a critical care physician [33]. The list of competencies, consisting of 129 competencies in 11 domains, was determined by a task force summoned by the Chinese College of Intensive and Critical Care Medicine (CCICCM). The list provided guidance to learners, their supervisors, and institutions in teaching and assessment. However, its length and the theoretical language employed present barriers to its direct use in learning needs assessments.

Previously reported learning needs assessments for Chinese medical professionals were predominately survey-based. Guo et al. conducted a 123-item learning needs survey among medical educators in China, aimed at identifying interest in various topics and perceived benefits and barriers of participating in faculty development programs [36]. Most study participants were hospital presidents or deans, potentially limiting the generalizability of these findings to the diverse learning needs of bedside interprofessional teams. The survey-based design could only reflect the responders’ perception of their learning preference, leaving unperceived learning needs uninvestigated.

In our study, the multimodal method had several strengths. First, Q method allowed us to study the typology of opinions within a heterogeneous group with a broad range of competencies, while traditional survey-based or test-based learning needs assessments are designed for groups with similar skillsets, such as medical students, nurses, or residents. We created EPAs to translate the theoretical concepts of ‘competencies’ into the core elements of practice. This list served as the foundation of the Q survey among interprofessional practitioners. Second, the mixed-method design was powerful in creating a complete, unbiased assessment. Chart audit, which is still rarely used for learning needs assessment of medical professionals, is especially useful in identifying unperceived learning needs, increasing awareness of practice weaknesses, and improving learning motivation. For example, chart audit in this study suggested the learners’ communication with families may not be adequate, information that could have been missed if the investigators only focused on learner responses. Chart audit also detected a suboptimal documentation rate of antimicrobial therapy discontinuation assessment, highlighting a need for better antimicrobial stewardship reflected by only one member of the learner group. Without the data from the chart audit, her valuable individual opinion could have been overlooked. By gathering subjective and objective data, we made our inferences about learning needs more robust.

The study has several limitations. First, to make the ranking activity convenient and feasible, the Q set only contained 20 EPAs. Recognizing the diverse nature of critical care practice, some learning needs could have been left ‘hidden’ if not properly described in the Q set. Moreover, although Q methodology is designed to study typology within a population, its semi-quantitative nature often results in multiple solutions of classification, while some opinions remain unclassifiable. Lastly, to maximize remote feasibility and limit cost we used chart audit as a surrogate for direct observation of participant daily clinical practices. This could have led to an underestimation of actual adherence to best practice based on documentation habits in the medical record.

## Conclusion

Multimodal learning needs assessment is feasible in interprofessional critical care groups and can be conducted remotely. Our methods identified our learners’ needs in various domains and effectively differentiated both divergent perceived and unperceived learning needs important for planning our educational intervention. These structured, yet flexible methods offer important tools to facilitate acceptance, engagement, and adoption of our customized critical care curriculum within the complex context of our learners’ practice environment. Our findings also indicated that addressing communication with patients and families may be challenging because of the difference in expectations and cultural norms. These results will help the investigators design an education program that includes both case-based discussions that provide a clinical context for discussions on common diagnostic and therapeutic challenges encountered in critical care, and remote simulation experiences to introduce a structured multidisciplinary rounding format to remind bedside teams to assess the necessity of devices and antimicrobial therapy. We are also partnering with our Chinese colleagues to better understand the best approach to discussions with families within their cultural and clinical context.

## Supplementary Information

**Additional file 1: Supplemental Table 1.** Johari window: different states of learning needs. **Supplemental Table 2.** Examples of learning needs assessment tools. **Supplemental Fig. 1.** Q survey, a ranking activity to express learning priorities. **Supplemental Table 3.** Patient baseline information on the day of admission.

## Data Availability

The data used for this research are available from the corresponding author on reasonable request and are subject to Institutional Review Board guidelines.

## Abbreviations

EPA: Entrustable Professional Activity
WHO: World Health Organization
CERTAIN: Checklist for Early Recognition and Treatment of Acute Illness and Injury
ICU: Intensive Care Unit
KTIP: Knowledge Translation into Practice
ACGME: Accreditation Council for Graduate Medical Education
PEEP: Positive End Expiratory Pressure
DVT: Deep Vein Thrombosis
CCICCM: Chinese College of Intensive and Critical Care Medicine
