# Supplementary material for: A novel multimodal needs assessment to inform the longitudinal education program for an international interprofessional critical care team
Source: BMC Med Educ. 2022 Jul 13;22:540. doi: 10.1186/s12909-022-03605-2 (PMC9281106; doi:10.1186/s12909-022-03605-2)
Supplement: Supplementary file 1 — Additional file 1: Supplemental Table 1. Johari window: different states of learning needs. Supplemental Table 2. Examples of learning needs assessment tools. Supplemental Fig. 1. Q survey, a ranking activity to express learning priorities. Supplemental Table 3. Patient baseline information on the day of admission. [file 12909_2022_3605_MOESM1_ESM.docx]

Supplemental Table 1 Johari window: different states of learning needs

|  | **Known to learners (perceived)** | **Unknown to learners**  **(unperceived)** |
| --- | --- | --- |
| **Known to others** | Open | Blind |
| **Unknown to others** | Hidden | Unknown |

Supplemental Table 2 Examples of learning needs assessment tools

| **Assessment Tools** | **Types of data**  **collected** | **Good for**  **determining** | **Advantages** | **Disadvantages** |
| --- | --- | --- | --- | --- |
| Informal discussion | Qualitative | Perceived needs | Convenient, inexpensive | Lack of methodological rigor |
| Questionnaires | Qualitative  Semi-quantitative | Perceived needs | Can sample large  groups | Validity is limited by quality of questions |
| Structured interviews | Qualitative | Perceived needs | Identify individual  learning needs | Interviewer biases |
| Chart audits | Quantitative | Unperceived needs | Identify areas of  weakness in a cohort of professionals | Requires effort and learners’ permission, only captures what is documented |
| Direct observation | Quantitative  and qualitative | Unperceived needs | Best method for assessing skills and performance | Time-consuming and costly, Hawthorne effect* |
| Test | Quantitative | Unperceived needs | Efficient, easy to conduct | Not real-life performance, not suitable for multidisciplinary groups |

*Hawthorne effect: the alteration of behavior by the subjects of a study due to their awareness of being observed.


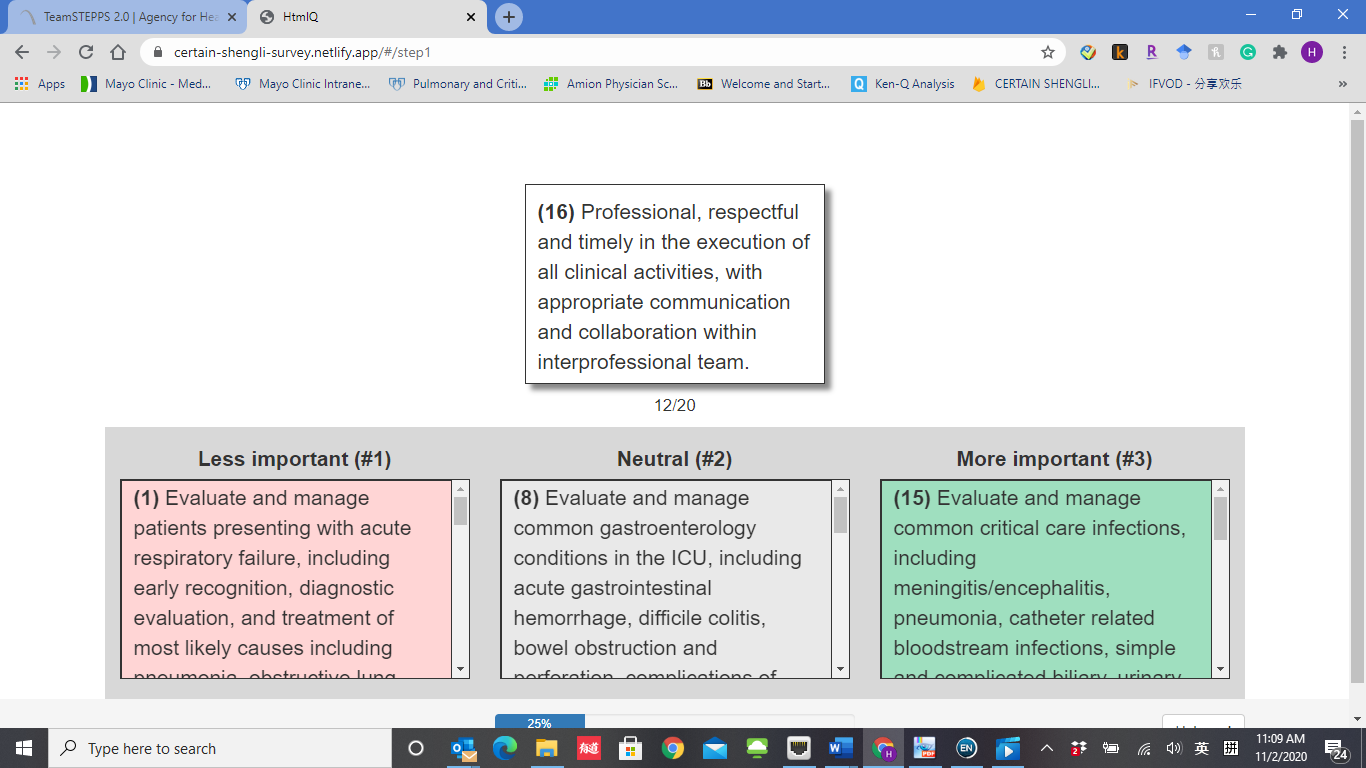


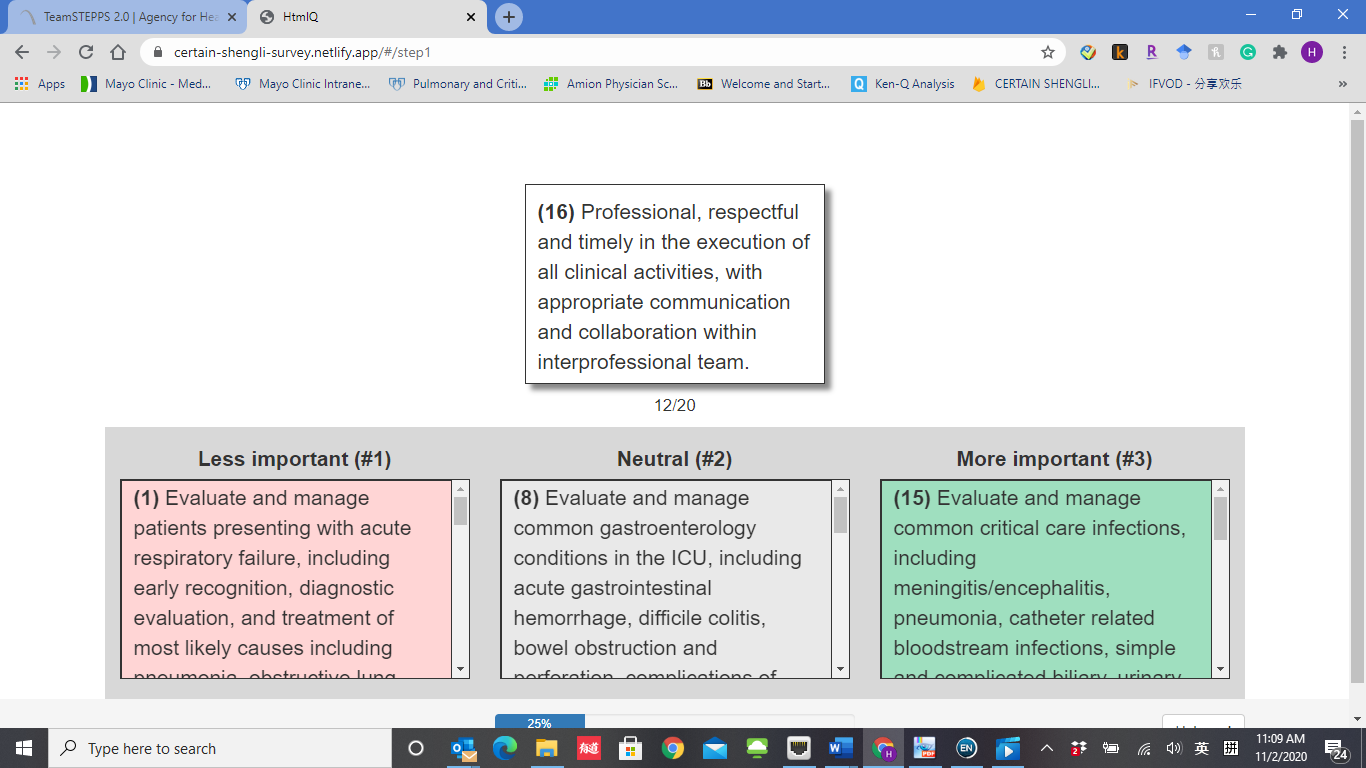


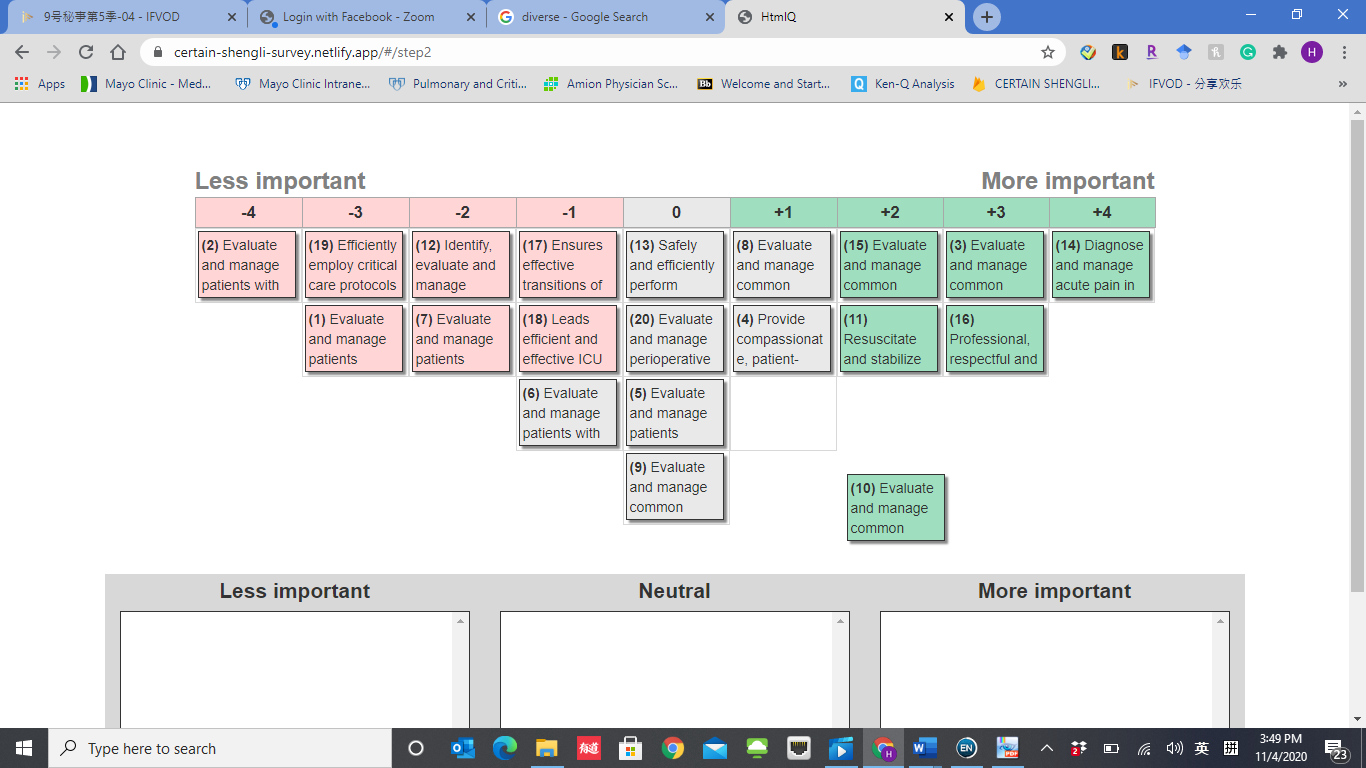


Supplemental Figure 1

Supplemental Figure 1 Q survey, a ranking activity to express learning priorities

Each individual in the learners’ group was asked to rank a set of cards with each EPA statement written on the card into ‘more important’, ‘less important’ and ‘neutral’ docks.

Then participants were asked to place the cards onto a pre-defined grid with a scale labeled on top based on their perceived importance of each EPA. The cards describing EPA statements most important for their training were placed on the right-most areas of the grid. The cards with EPAs less important were placed on the left side of the grid. The cards close to the middle of the field represented a neutral or undetermined perception by the participant. In this study, the statement that was placed at the ‘most important’ end of the distribution received a score of +4, the next two statements received +3, the next two statement received +2, and so forth, all the way down to the statement that was considered ‘least important’, which received a score of -4. Statements placed in the middle of the grid were assigned scores of 0.

Supplemental Table 3 Patient baseline information on the day of admission

| **Age median (IQR*)** | 74(62,81.5) |
| --- | --- |
| **Gender, female, n(%)** | 29(34.1) |
| **Hospital admission source, n(%)** |  |
| Home | 80(94.1) |
| Nursing home | 0(0) |
| Outside hospital | 4(4.7) |
| others | 1(1.2) |
| **Limitation of life-support measures, n(%)** | 4(4.7) |
| **Apache II score within 6 hours,**  median (IQR), N=80 | 22(16,29) |
| **Comorbidities, n(%)** |  |
| Congestive heart failure | 25(29.4) |
| Hypertension | 17(20.0) |
| Pulmonary circulation disorders | 14(16.5) |
| Diabetes | 13(15.3) |
| Renal failure | 12(14.1) |
| Arrhythmia | 12(14.1) |
| Obesity | 5(5.9) |
| Cancer | 4(4.7) |
| Liver diseases | 3(3.5) |

*IQR: Interquartile range
